# Lurasidone an Effective Alternative for the Treatment of Irritability Associated With Autism Spectrum Disorder

**DOI:** 10.7759/cureus.12360

**Published:** 2020-12-29

**Authors:** Mayank Gupta, Gary Hoover

**Affiliations:** 1 Psychiatry, Lake Erie College of Osteopathic Medicine, Erie, USA; 2 Psychiatry and Behavioral Sciences, Clarion Psychiatric Center, Clarion, USA; 3 Physical Medicine and Rehabilitation, Allegheny Health Network, Pittsburgh, USA

**Keywords:** lurasidone, autism

## Abstract

The key objective to demonstrate the use of Lurasidone in children or adolescents with autism spectrum disorders (ASD) and add further insights into its use in clinical practice with youth. We present four cases treated in the inpatient settings with unique challenges and their treatment course to address the unique clinical practice challenges. There is a dearth of psychopharmacologic options to treat irritability and affective symptoms in children and/or adolescents with autism spectrum disorders. Although recent research has yielded mixed results, there is still a not clear consensus on its effectiveness. Based on our experience, we found Lurasidone was an effective alternative due to its unique psychopharmacologic profile. It was well tolerated without any side effects, and its efficacy was more observed at higher doses.

## Introduction

There are no established pharmacologic treatments for autism spectrum disorder (ASD) [1]. Currently, Risperidone [2] and Aripiprazole [3] are two second-generation antipsychotics (SGA) [4] approved by the Food and Drug Administration (FDA) for irritability and disruptive behaviours associated with ASD. A meta-analysis of randomized controlled trials (RCT) confirmed that Risperidone [5] and Aripiprazole have been most effective with large effect size [6]. However, adverse effects like weight gain, metabolic changes, sedation and increased prolactin levels have been reported as major concerns [7-8]. Lurasidone (Latuda) is a newer atypical antipsychotic that was FDA approved in 2010 to treat schizophrenia and bipolar depression [9] in adults. It is efficacy [10], and safety [11] profile has been established [12]. It primarily acts on D2 and 5HT2A receptors [13]. It has a favourable metabolic profile, less likely to cause weight gain [14] and no effect on QTc [15]. It has less affinity for postsynaptic α1‐adrenergic receptors and less likely to increase prolactin. Its minimal effective dose is 40mg and is more effective if the dose is doubled after weeks of no response in schizophrenia [16].In this case series, we report the use of Lurasidone for irritability and mood symptoms associated with an autism spectrum disorder.

## Case presentation

Case 1

The patient was a 16-year-old Caucasian male diagnosed with an autism spectrum disorder, oppositional defiant disorder (ODD), and mood disorder unspecified. He was admitted to the inpatient unit after an outburst which included physical aggression and property destruction. The patient had not taken any of his medications during the previous several weeks leading up to the admission. The patient denied any history of sexual or physical abuse. He also denied any suicidal ideations or behaviours. He lived at home with his parents and adolescent brother. A family history of bipolar disorder in his mother and an older brother was diagnosed with attention deficit hyperactive disorder and ASD. On examination, he was tearful, had flat affected and marked irritability. He demonstrated poor insight and judgment. The patient reported difficulty managing his anger and demonstrated poor social interactions and relationships, which was evident from the lack of friends and his preference for solitary activities. He also had multiple prior instances of violence and property destruction. The patient admitted to rapid mood swings, poor sleep, and racing thoughts. He stated that he had repeatedly threatened his peers when he did not get his way and often acted upon his impulses. He also admitted to poor adherence to Chlorpromazine due to its sedating side effects. He denied any hallucinations and drug use.

The patient’s home medication list included albuterol as needed, Cogentin 0.5mg 3 times per day, BuSpar 10mg twice a day, Chlorpromazine 100mg at bedtime and 25mg three times a day, Desmopressin 0.2mg at bedtime, Flovent 110mcg twice a day, and Eskalith 450 mg twice a day. The patient was tried on Risperidone in the past, but his outpatient provider stopped it due to excessive weight gain and sedation. After admission, he was started on Aripiprazole 5mg PO AM daily, Guanfacine 0.5mg PO daily, and Lithium carbonate 300mg PO twice daily. The patient reported sedation due to the Aripiprazole and refused informed consent to continue it. Therefore Aripiprazole was discontinued for Latuda 20mg PO every morning and continued on lithium carbonate Extended Release (ER) 300mg PO twice per day. His initial lithium level was 0.5. The dose of Latuda was gradually increased over one week to 80mg PO every morning, and Lithium carbonate was increased to 450mg PO twice a day. After the dose was increased, his second Lithium levels were 0.7. Guanfacine was also increased to 1mg PO at night for impulsivity. At the beginning of the treatment period, the patient was isolative, had difficulty interacting with his peers, and demonstrated paranoid ideations about his peers. He was impulsive and agitated. As his treatment progressed, the patient’s mood improved, and he became less irritable. He initially remained isolative and continued to have poor interactions with peers. Towards the end of his ten days of inpatient stay, the patient started participating in group activities and had some positive, albeit superficial, social interactions with his peers. Upon discharge, he continued to have some social interaction difficulties, though they were not as marked as before initiation of therapy. He also continued to fixate on topics and have rigid thoughts.

Case 2

The patient was a 15-year-old Caucasian female diagnosed with autism spectrum disorder and intermittent explosive disorder. She was admitted to the the inpatient unit with suicidal ideations. She had a longstanding history of significant problems with social relationships, anxiety, and impulse control. She reported average academic performance but had difficulties with integrating into a social environment. She also reported a multitude of worries, including not being socially accepted, not performing well in school, and being perceived as socially inadequate. There were no developmental delays reported in this patient. However, there were reports of fainting spells and neonatal jaundice during the patient’s early neonatal life. She denied any previous history of inpatient psychiatric hospitalization but reported receiving outpatient psychiatric treatment. She reported a previous suicide attempt but denied any past medical history. Her medications upon admission were Lexapro 10mg daily and Aripiprazole 2 mg once daily. She denied any substance abuse history. Family history was negative for mental health problems and suicide attempts, but positive for alcohol abuse. On examination, this patient was isolative and impulsive. She reported poor sleep, hopelessness, and racing thoughts. She described her mood as depressed. She reported a long history of being bullied. She also admitted to suicidal ideations with a plan and intent. She denied experiencing hallucinations and also denied any history of physical or sexual abuse. Patient and family requested medication change since she experienced akathisia on Aripiprazole. This patient was started on Lurasidone 20mg PO every night for mood and Guanfacine 0.5mg PO twice daily for impulsivity after admission. This dose of Lurasidone was titrated up to 60mg PO every night. At the beginning of her treatment, the patient demonstrated poor social interactions, emotional dysregulation, and interpersonal relationships problems. The patient reported a noticeable improvement in her mood a few days after treatment began. After nearly two weeks of treatment, suicidal ideations were no longer present. The patient participated more in group activities and demonstrated positive interactions with her peers as the treatment progressed. She also reported that the Lurasidone helped to eliminate negative thinking. The patient reported no side effects from the medication and stated that it was helping her with sleep.

Case 3

The patient was an 18-year-old Caucasian female with a history of autism spectrum disorder, depressive disorder NOS, intermittent explosive disorder, Attention deficit hyperactivity disorder (ADHD) and learning disorder. She was admitted to an inpatient unit for suicidal ideations with a plan. This patient had several previous admissions to inpatient psychiatric centres and was part of an outpatient program for her mental illness. She had a longstanding history of poor social support, difficulties with social communication, and impulsive behaviours. She also had a history of living in a detention centre and foster care during childhood. At the time of admission, she was living with a family friend who served as her caretaker. She reported that her family history was significant for mental illness in several immediate family members but did not specify the disorders they suffered. She denied substance abuse but reported a history of physical and sexual abuse. She also had a history of multiple suicide attempts and self-harming behaviours. Her medication list at the time of admission included perphenazine 4mg daily, microgestin FE 1.5/30 daily, Ventolin inhaler two puffs every 4 hours, Lamotrigine ER 200 mg twice Quetiapine 150mg AM and 300mg HS, and Vistaril 25 mg three times daily. In the past, she had been on Aripiprazole which was stopped due to weight gain. On examination, her mood was irritable, and her affect was constricted. However, she was cooperative throughout the interview. She appeared to have poor social skills, especially with eye contact and social reciprocity. She demonstrated poor insight and judgment. She reported anxiety and agitation. She also reported feelings of hopelessness. She was started on Lurasidone 20mg PO AM and Lithium 150mg twice daily on admission for her mood. She has also prescribed Guanfacine 0.5 mg PO twice daily for impulsivity. Her dose of Lurasidone was increased to 60 mg PO AM daily a week after admission, and her Guanfacine was increased to 0.5mg PO three times daily. She reported that her mood had improved since the admission but reported some gastrointestinal symptoms. Lithium was discontinued to alleviate gastrointestinal distress. She was also given ondansetron 4mg PO every 6 hours for nausea. She displayed gastroesophageal reflux disease symptoms and was given Pantoprazole 40mg PO twice daily and Tagamet HB 200mg PO three times daily. She also received a dietary consult due to a history of lactose intolerance. At the beginning of her treatment, the patient was generally withdrawn, often isolating her room and not participating in group activities. She had multiple somatic complaints during her stay, but appropriate workups did not yield any significant findings. As her treatment progressed, she slowly began including herself in group activities and occasionally interacting with peers. Her mood improved after two weeks of treatment. She also showed some improvement in social reciprocity and became less isolative.

Case 4

Patient was a 14-year-old Caucasian male who was hospitalized after displaying violent behaviour towards his guardian. The guardian reported that the patient behaved in an out of control manner, which included violent outbursts and property destruction. The patient reported that he had mood problems in the months leading up to his admission and that his medication regimen at the time was not sufficient. He reported mood swings and paranoid ideations. He also admitted to difficulties paying attention, aggression, impulsivity, irritability, defiance, and mood dysregulation. He had a history of multiple inpatient psychiatric hospitalizations at different institutions. His past psychiatric history included a diagnosis of autism spectrum disorder, ODD and ADHD. At the time of admission, his medications included Dexmethylphenidate 10mg twice daily, Chlorpromazine 25mg three times daily, clonidine 0.1 mg once daily, and albuterol two puffs every 4-6 hours. His medical history included asthma, tympanostomy tubes, tonsillectomy, and adenoidectomy. The patient denied any history of head trauma. The patient admitted to poor peer relationships and difficulties with academic performance. He also reported a history of sexual abuse. Family history was significant for bipolar disorder in his mother and substance abuse in both his mother and father. The patient was living with his grandmother and three siblings at the time of admission. Upon evaluation, he was unkempt and dishevelled. He displayed psychomotor agitation throughout the assessment and spoke with an increased rate and volume of speech. There were no language difficulties, although the patient avoided eye contact and had difficulty establishing rapport. He had poor social skills and pragmatics. His thoughts were tangential, his speech was pressured, and he showed a flight of ideas. His long- and short-term memory were intact, his intelligence was average, and his insight and judgment were poor.

The admitting diagnoses were disruptive mood dysregulation disorder, ADHD, combined type; intermittent explosive disorder, ODD, and autism spectrum disorder. He was started on Lurasidone 20mg PO AM daily and Lithium 150mg PO twice daily. He was also started on Guanfacine 0.5mg PO twice daily for ADHD. The Lurasidone was slowly titrated up to 80mg PO daily. The lithium was changed to lithium carbonate ER 450mg PO twice daily to reach therapeutic levels. The patient’s mood stabilized after two weeks of treatment on this regimen, and he reported no side effects. He also participated in more group activities and showed improvement in interactions with his peers throughout his treatment. He was making better eye contact and appropriately initiating conversations. He denied any anxiety, depression, suicidal ideations, homicidal ideations, or irritability at the time of discharge. He reported that his impulses were well-controlled.

## Discussion

In these cases, off label use of Lurasidone has been useful for improving irritability and disruptive mood associated with an autism spectrum disorder. These patients showed improvements in social interactions, such as becoming less withdrawn and seeking out peers and staff for conversation. There were also no reports of side effects such as weight changes or sedation that can occur with other atypical antipsychotics. One of our patients did have gastrointestinal complaints, but those seemed to improve once her lithium carbonate was discontinued, and her diet was changed. The rationale to consider Lurasidone was that these patients have failed to respond to the approved medications or refusal to continue due to undesired side effects. One of the weaknesses was concomitant use of Lithium in two cases that have efficacy in mood disorders and could be a confounding factor. There were no drug interactions with the medication regimen, and most of the patients tolerated it well. Also, most of these patients were admitted to multiple psychotropic agents, and the treatment goal was to reduce the number of medication used to address the core symptoms. Since 2014, there have been case reports [17] which suggest positive response with Lurasidone use. However, a placebo-controlled RCT [18] demonstrated no change in irritability. Lurasidone is still used off label [19] to address these symptoms.

## Conclusions

It was also noted that a more robust response was evident at doses higher than 60mg. Many patients have been tried on both approved medications, Risperidone and Aripiprazole, without desired outcomes in clinical practice. Therefore, there is always a need for other options. In this case series, Lurasidone has shown promise in treating the behavioural and mood symptoms related to autism spectrum disorders. Further studies, such as randomized-controlled trials and longitudinal studies, are needed.
